# F75. MEDICATION ADHERENCE AND DISCONTINUATION IN PATIENTS WITH SCHIZOPHRENIA TREATED WITH ARIPIPRAZOLE ONCE-MONTHLY LONG-ACTING INJECTABLE VERSUS THOSE TREATED WITH ORAL ANTIPSYCHOTICS

**DOI:** 10.1093/schbul/sby017.606

**Published:** 2018-04-01

**Authors:** Mallik Greene, Tingjian Yan, Eunice Chang, Ann Hartry, Beth Pulasky

**Affiliations:** 1 Otsuka Pharmaceutical Development & Commercialization, Inc. (OPDC); 2 Partnership for Health Analytic Research, LLC; 3 Lundbeck

## Abstract

**Background:**

Adherence to antipsychotic treatment is essential in treating schizophrenia symptoms and in preventing costly relapse. This study aimed to compare medication adherence and discontinuation in patients with schizophrenia treated with aripiprazole once-monthly long-acting injectable antipsychotic (LAI; AOM 400) to those who changed to a different oral antipsychotic.

**Methods:**

This retrospective cohort analysis used the Truven Health Analytics MarketScan® Medicaid, commercial, and supplemental Medicare claims databases. In patients ≥18 years old with schizophrenia, two mutually exclusive cohorts were created: the AOM 400 cohort, patients who initiated AOM 400 between 01/01/2013-06/30/2015 (the ID period); and the oral cohort, patients who changed to a different oral antipsychotic during the ID period. AOM 400 or new oral therapy initiation was the index date. Patients were followed for ≥1 year. Primary outcome measures were adherence (proportion of days covered [PDC]) during 1-year post-index and index medication discontinuation (gap ≥60 days) during entire follow-up. Cox regression and linear regression models were used to estimate risk of discontinuation and PDC, respectively, adjusting for demographic and clinical characteristics, insurance type, baseline medication, and baseline ED visits or hospitalizations.

**Results:**

The study sample consisted of 408 (10.8%) AOM 400 patients and 3,361 (89.2%) oral antipsychotic patients. AOM 400 patients had better medication adherence (adjusted mean PDC: 57.0% vs. 47.6%, p<0.001) than the oral cohort. Sixty-three percent of AOM 400 patients were partially (PDC 40%-79%) to fully adherent (PDC >80%) vs. 51.1% of oral antipsychotic patients (p<0.001). AOM 400 patients also had a lower medication discontinuation rate (75.2% vs. 85.0%; p<0.001) within 1 year. Median time to discontinuation of AOM 400 was 193 days vs. 89 days for oral antipsychotics (p<0.001). In the Cox model, oral antipsychotic patients discontinued their index treatment at a higher rate than AOM 400 patients (hazard ratio: 1.45; p<0.001).

**Discussion:**

This real-world study suggests that patients with schizophrenia initiating AOM 400 had better medication adherence and lower discontinuation risk than patients who changed to different oral antipsychotics.

